# Assessing competency in Evidence Based Practice: strengths and limitations of current tools in practice

**DOI:** 10.1186/1472-6920-9-53

**Published:** 2009-08-06

**Authors:** Dragan Ilic

**Affiliations:** 1 Monash Institute of Health Services Research, Monash University, Clayton, VIC 3168, Australia

## Abstract

**Background:**

Evidence Based Practice (EBP) involves making clinical decisions informed by the most relevant and valid evidence available. Competence can broadly be defined as a concept that incorporates a variety of domains including knowledge, skills and attitudes. Adopting an evidence-based approach to practice requires differing competencies across various domains including literature searching, critical appraisal and communication. This paper examines the current tools available to assess EBP competence and compares their applicability to existing assessment techniques used in medicine, nursing and health sciences.

**Discussion:**

Only two validated assessment tools have been developed to specifically assess all aspects of EBP competence. Of the two tools (*Berlin *and *Fresno *tools), only the *Fresno *tool comprehensively assesses EBP competency across all relevant domains. However, both tools focus on assessing EBP competency in medical students; therefore neither can be used for assessing EBP competency across different health disciplines. The Objective Structured Clinical Exam (OSCE) has been demonstrated as a reliable and versatile tool to assess clinical competencies, practical and communication skills. The OSCE has scope as an alternate method for assessing EBP competency, since it combines assessment of cognitive skills including knowledge, reasoning and communication. However, further research is needed to develop the OSCE as a viable method for assessing EBP competency.

**Summary:**

Demonstrating EBP competence is a complex task – therefore no single assessment method can adequately provide all of the necessary data to assess complete EBP competence. There is a need for further research to explore how EBP competence is best assessed; be it in written formats, such as the *Fresno *tool, or another format, such as the OSCE. Future tools must also incorporate measures of assessing how EBP competence affects clinician behaviour and attitudes as well as clinical outcomes in real-time situations. This research should also be conducted across a variety of health disciplines to best inform practice.

## Background

### What is competence and why is it important?

Competence can broadly be defined as a concept that incorporates a variety of domains including knowledge, skills and attitudes. [1] Health professionals may demonstrate overall competence in their relevant discipline via a four step process including; (1) knowledge, (2) competence (specific to the task), (3) performance, and (4) action. [2] Apart from knowledge, skills and attitudes, competence also incorporates a health professional's problem solving skills (e.g. ability to critically think and apply clinical reasoning) and ability to work as a team member and communicate effectively, both in a written and verbal format. [3] Assessing competences can focus on any one of these domains.

### What is Evidence Based Practice (EBP) competence?

Evidence based practice (EBP) involves making clinical decisions informed by the most relevant and valid evidence available. [4] EBP has been described as the integration of clinical expertise and patient values with the best available research evidence. [4] Clinical expertise draws on the health professional's clinical skills and past experience to identify and treat each patient's individual circumstance. Patient values encompass the personal concerns, expectations, cultural influences and characteristics of individuals during the clinical encounter. The best research evidence draws on the highest quality of clinically related research. The integration of these three elements increases the potential for positive health outcomes.

EBP requires the health professional to apply the best available evidence to assist with their clinical decision making. [4] The practice of EBP consists of the following key steps;

1. Converting clinical scenarios into a structured answerable question,

2. Searching the literature to identify the best available evidence to answer the question,

3. Critically appraising the evidence for its validity and applicability, and

4. Applying the results of the appraisal into clinical practice

5. Evaluation/assessment of the EBP process. [4]

Each step of the EBP process requires a different level of knowledge and skill (i.e. competence).

• Step 1 requires knowledge to construct a question using the PICO mnemonic,

• Step 2 requires the acquisition and application of literature searching skills across a variety of databases,

• Step 3 requires a certain level of expertise in epidemiology and biostatistics, and

• Step 4 requires an ability to synthesise and communicate the results to relevant parties (i.e. health professionals, patients).

• Step 5 requires the health professional to evaluate the EBP process and assess its impact within the clinical context in which it was implemented.[5]

### Has assessment of EBP competence previously been investigated?

A systematic review published in 2001 investigated the effects of teaching EBP skills to health professionals with respect to their EBP competence. [6] It identified one randomised controlled trial (RCT), which concluded that teaching EBP skills in a post-graduate environment increased participants' EBP knowledge and skills. However, a limitation of that RCT was that no validated assessment tool was applied to distinguish the effects of pre/post training in EBP. Rather, participants were asked to complete a 'self-assessment' of their EBP competencies. Self-review is a subjective form of assessment, with participants often factoring other variables that they perceive may have influenced their performance, thereby skewing the actual performance and outcome. [7] Participants are also prone to experiencing recall bias, whereby they may believe that their baseline ability was much poorer than it actually was, therefore increasing their perceived improvement following the training intervention.

## Discussion

Few validated assessment tools have been developed to assess EBP competence. Assessment tools used to assess EBP competence have primarily focussed on medical students and graduates. [8] The majority of these assessment tools have been self reports and learner satisfaction questionnaires – both of which are limited in their use in assessing EBP competence as previously explained. [9-11] A recent systematic review appraised instruments for evaluating EBP teaching. [12] It identified 104 unique instruments, most of which were administered to medical students and postgraduate trainees. These instruments aim to evaluate EBP competence of students/trainees, effectiveness of EBP curricula and student/trainee behaviour. It identified that the majority of instruments predominantly focused only on one aspect of EBP (critical appraisal). The *Fresno *and *Berlin *assessment tools were the only instruments to evaluate all steps of the EBP process; with both containing measured psychometric properties, objective measured outcomes and established validity and reliability references for individuals. [12-14]

### The Berlin assessment tool

The *Berlin *assessment tool measures medical professionals' EBP competence (skills and knowledge). [14] It was constructed by a panel of EBP experts and validated in a group of medical health professionals attending a course on EBP. The *Berlin *tool consists of 15 multiple choice questions (MCQs), which primarily focused on assessing the participant's epidemiological skills and knowledge. The EBP competencies of participants were compared to a 'control' group of medical professionals at the conclusion of the course. The *Berlin *tool was able to reliably distinguish expertise between the two groups. Although the *Berlin *tool is described as a tool that can assess EBP competence, it only assesses one component of EBP ('Step 3' of the EBP process). It contains no assessment of the other three key steps needed to demonstrate complete competence in EBP. Similarly, it has only been designed to assess EBP competence in medicine – it does not assess EBP competence across other health disciplines (e.g. nursing, allied health etc). Therefore, the *Berlin *tool can only at best truly assess one component of EBP competence.

### The Fresno assessment tool

The *Fresno *assessment tool also measures medical professionals' EBP competence (skills and knowledge). [13] The *Fresno *tool consists of two clinical scenarios with open ended questions. Participants are required to complete the four key steps of EBP process in order to adequately answer the open ended questions relating to the clinical scenarios. The *Fresno *tool has been validated with medical residents and has shown to have good inter-rater reliability, since it requires expert knowledge to assess open ended answers. The *Fresno *tool is the only standardised, objective measure of EBP competence currently available, since it measures the participants' knowledge and skill across the four key EBP steps. It requires the participant to demonstrate their knowledge, competence, performance and action across all four components to successfully demonstrate EBP competence. [2] Although the *Fresno *tool assesses complete EBP competence, it is limited in its applications as it has only been developed for use in medicine. Therefore, it cannot be used to assess EBP competence in other health disciplines (e.g. nursing, allied health).

### What other assessment tools could be used to assess EBP competence?

Written items, such as Extended Matching Questions (EMQs) and MCQs, are best utilised to assess the learner's core clinical knowledge [15]. These assessment tools may be useful in assessing one aspect of EBP competence (e.g. step 3), as is the case with the *Berlin *test; however, they are not suitable if competence across all four EBP domains is sought. Although there are several papers providing MCQs and EMQs to assess EBP knowledge,[16] no literature currently explores the validity of using MCQs or EMQs as an assessment tool for EBP competence. Several self-directed, continuing education exercises such as the PEARLS (Presentations of Evidence Abstracted from the Research Literature for the Solution of Real Individuals' Clinical Problems) exercise provide clinicians with a formative method of assessing their EBP competence. [17,18] These exercises also provide practicing clinicians with the opportunity to integrate the principles of EBP in their daily clinical environment. However, little research has been done to ascertain the psychometric properties, and established validity and reliability references for such tools.

The Objective Structured Clinical Exam (OSCE) has been demonstrated as a reliable and versatile tool to assess student clinical competencies, practical and communication skills. [19-22] Assessing competence in EBP can be difficult due to the various cognitive skills and knowledge that must be performed. However, the OSCE has great scope to adequately test student competency for various reasons. The OSCE simulates 'real-life' situations that the student may encounter in the clinical environment. Recently, several studies have published preliminary results exploring the value of assessing EBP competency via OSCEs. [23-27] All of the studies reported very good construct validity and inter-rater reliability. However, few assess all four components within the OSCE framework and all studies were conducted with undergraduate medical students; thereby limiting the generalisability of the results. Additionally, none of the studies incorporated a validated tool for assessing EBP competence; with participants were assessed according to a pre-determined check list, or on a Likert scale. [23-27]

### How is EBP competence assessed across health disciplines?

Due to the lack of data in the current literature it is not possible to compare how EBP competence is assessed across difference health disciplines. The *Berlin *and *Fresno *tools have both been validated as tools to assess EBP competence within medicine. Another version of the *Fresno *tool is currently being developed to assess EBP competence in other health disciplines. However, apart from that development no other assessment tools, or studies investigating assessing EBP competence in disciplines other than medicine, have been published.

It has been little more than a decade since the notion of integrating evidence into clinical decision making was proposed and the field of EBP first developed. [4] The past decade has seen tremendous growth in the field, with institutions such as the Cochrane Collaboration, and methodologies, including the systematic review, now widely accepted. Whilst tremendous effort has been put forth into the development of EBP methodologies and teaching EBP competencies, relatively little research has been performed on these topics. [8]

There is a dearth of literature exploring how EBP should best be taught (e.g. lectures, tutorials, case based presentations and journal clubs) – therefore it is difficult to ascertain how EBP competencies should be assessed. Many of the studies published on teaching EBP have focussed on methods to impart new knowledge. In doing so researchers have struggled to define the specific changes they wish to achieve in implementing their EBP teaching interventions (e.g. knowledge, behaviour, or both). Until recently these studies have relied on assessing EBP related competencies on self reports and ad-hoc evaluations, rather than validated assessment tools such as the *Berlin *and *Fresno *tools. Even with the advent of the *Berlin *and *Fresno *tools, no new studies, apart from the original papers, have adopted their use in assessing EBP competency.

### Avenues for future research

Further research needs to be conducted across a variety of areas to comprehensibly explore assessment of EBP competence. Further development on specific assessment tools, such as the *Fresno *tool, is required so that it can be applied across various health disciplines. Developing an OSCE version of the *Fresno *tool would further enhance its ability to assess participants' communication skills in EBP. An OSCE version of this tool would provide greater scope to assess specific EBP competencies (such as searching the literature online) in a restricted timed environment that mimics the real time situation that most clinicians will experience.

Impact on clinical behaviour and outcome is an important measure of EBP. The *Fresno *and *Berlin *tools assess all four domains of EBP however; neither assesses the fifth element of EBP – evaluating/assessing the effectiveness of the EBP process. One method posed for evaluating the EBP process is conducting an audit of clinical processes and outcomes. Such a process would entail comparing actual practice, as a result of adopting an EBP approach, to a standard of practice. [28] A simple method of conducting such an audit may include incorporating an activity diary to document activities directly related to EBP, such as online searching or critical appraisal. [29] Few current instruments assess changes in behaviour and attitudes in great depth, with none exhibiting acceptable levels of validity. [12] The use of such diaries should also be explored as a method for evaluating any changes in attitude and/or behaviour directly related to EBP.

Whilst achieving a high level of EBP competence might be desirable for many health professionals, others might prefer achieving a high level in only certain domains of EBP. This divergence in needs has lent support to adopting a framework for evaluating teaching methods for EBP. [30] Such a framework ponders evaluating EBP competence according to the need of the learner. A busy clinician may only wish to utilise pre-appraised information, hence it may be appropriate not to evaluate step 3 of the EBP process. Further research is needed to identify whether the *Fresno *and *Berlin *tools can be modified and integrate other essential aspects to evaluation, such as clinical audit and EBP competence according to needs. It is also necessary to explore how pragmatic such an integrated approach may be across several health disciplines.

Summary

• There is a current dearth of evidence exploring the best methods of assessing EBP.

• The *Fresno *tool currently is the most appropriate tool to assess EBP competence.

• Further development of the *Fresno *tool is needed to accommodate assessment of EBP competence across a variety a health disciplines.

• Demonstrating EBP competence is a complex task – no single assessment method can adequately provide all of the necessary data to assess complete EBP competence.

• Future tools must incorporate measures of assessing how EBP competence affects clinician behaviour and attitudes as well as clinical outcomes in real-time situations.

## Competing interests

The author declares that they have no competing interests.

## Authors' contributions

DI conceived and drafted the manuscript.

## About the Author

DI is a Senior Lecturer in Evidence Based Clinical Practice at the School of Public Health & Preventive Medicine in Monash University. He co-ordinates teaching of EBP across undergraduate and graduate levels.

## Pre-publication history

The pre-publication history for this paper can be accessed here:


